# Synthesis of a tubugi-1-toxin conjugate by a modulizable disulfide linker system with a neuropeptide Y analogue showing selectivity for hY1R-overexpressing tumor cells

**DOI:** 10.3762/bjoc.15.11

**Published:** 2019-01-10

**Authors:** Rainer Kufka, Robert Rennert, Goran N Kaluđerović, Lutz Weber, Wolfgang Richter, Ludger A Wessjohann

**Affiliations:** 1 Department of Bioorganic Chemistry, Leibniz Institute of Plant Biochemistry, Weinberg 3, D-06120 Halle (Saale), Germany; 2 OntoChem GmbH, Blücherstr. 24, D-06120 Halle (Saale), Germany; 3 TUBE Pharmaceuticals GmbH, Leberstr. 20, A-1110 Vienna, Austria

**Keywords:** drug targeting, neuropeptide Y, PDC, peptide–drug conjugate, targeted tumor therapy, tubugi, tubulysin A, Ugi reaction

## Abstract

Tubugi-1 is a small cytotoxic peptide with picomolar cytotoxicity. To improve its cancer cell targeting, it was conjugated using a universal, modular disulfide derivative. This allowed conjugation to a neuropeptide-Y (NPY)-inspired peptide [K^4^(C-βA-),F^7^,L^17^,P^34^]-hNPY, acting as NPY Y1 receptor (hY1R)-targeting peptide, to form a tubugi-1–SS–NPY disulfide-linked conjugate. The cytotoxic impacts of the novel tubugi-1–NPY peptide–toxin conjugate, as well as of free tubugi-1, and tubugi-1 bearing the thiol spacer (liberated from tubugi-1–NPY conjugate), and native tubulysin A as reference were investigated by in vitro cell viability and proliferation screenings. The tumor cell lines HT-29, Colo320 (both colon cancer), PC-3 (prostate cancer), and in conjunction with RT-qPCR analyses of the hY1R expression, the cell lines SK-N-MC (Ewing`s sarcoma), MDA-MB-468, MDA-MB-231 (both breast cancer) and 184B5 (normal breast; chemically transformed) were investigated. As hoped, the toxicity of tubugi-1 was masked, with IC_50_ values decreased by ca. 1,000-fold compared to the free toxin. Due to intracellular linker cleavage, the cytotoxic potency of the liberated tubugi-1 that, however, still bears the thiol spacer (tubugi-1-SH) was restored and up to 10-fold higher compared to the entire peptide–toxin conjugate. The conjugate shows toxic selectivity to tumor cell lines overexpressing the hY1R receptor subtype like, e.g., the hard to treat triple-negative breast cancer MDA-MB-468 cells.

## Introduction

Until recently, the medication of tumor diseases was primarily based on more or less unspecific chemotherapeutics and the corresponding combination therapies [1–5]. However, severe impairments of normal, non-transformed tissues caused by widespread off-target effects have limited the therapeutic benefits of many classical chemotherapeutics [6]. Within the last two decades, progress in basic research on the biochemical, molecular biological and medicinal aspects of a broad range of tumor diseases, as well as progress in drug development technologies provided the basis for a fundamental paradigm shift in cancer treatment, away from non-selective cytotoxic chemotherapeutics towards specifically tumor-targeting therapeutics [7–8]. Such targeted therapeutics are able to address transformed cells selectively by recognition of disease-associated membrane structures, e.g., dysregulated membrane proteins, or by modulation of metabolic or regulatory characteristics that are specific or at least differential for tumor cells. Members of one prominent novel class of targeted anticancer drugs that has been developed over the last years are antibody–drug conjugates (ADCs) [9–11]. Due to their high antibody-mediated target specificity, ADCs are designed for selective treatments of tumor cells with very potent, mostly cytotoxic drug molecules while avoiding or at least limiting the off-target toxicity that would be characteristic for the stand-alone cytotoxic drugs. Currently, four therapeutic ADCs are approved, e.g., with brentuximab vedotin and trastuzumab emtansine as the first ones on the market. However, many other ADC development projects are in clinical trials [12–13].

More recently, peptide–drug conjugates (PDCs) have been recommended as targeted therapeutics [14–15]. While sharing the ADCs’ therapeutic concept of targeted and highly selective drug addressing to the diseased cells, PDCs are smaller in size – which may improve tissue and cell permeability, allows a more flexible and cost-efficient production, and in many cases small peptides are less antigenic [16].

Generally, a useful PDC must exhibit at least four major skills that are all required for the selective and potent treatment of, for instance, cancer cells: (1) a sufficient in vivo half-life, ideally hours to days, to reach the diseased cells with a high portion of intact PDC; (2) a selective conjugate binding to a specific target molecule, e.g., a cell-surface receptor, that is characteristic for the diseased cells; (3) a fast and efficient but target-dependent binding, or better internalization, of the PDC into targeted cells; and (4) the efficient cleavage of the linker structure and, thereby, efficient liberation of the drug molecule from the conjugate at or within the diseased cell, resulting in an efficient intracellular drug dose, ideally killing the tumor cells.

PDCs have been demonstrated to achieve efficient and target-specific delivery of conjugated payloads, primarily highly potent toxins or chelated radiotracers, to tumor cells. In that context, the peptide moiety of the PDC is responsible for the selective targeting of the conjugate towards a specific molecular structure that has been identified to be characteristic for a diseased state of cells and tissues. Particularly G protein-coupled receptors (GPCRs) that are endogenously activated by agonistic peptide or protein ligands can be suitable target structures. Many peptide or protein ligand receptors have been associated with various diseases, e.g., cancer malignancies [17]. Amongst the GPCRs, the neuropeptide Y (NPY) receptor family comprises four closely related receptor subtypes in human (hY1R, hY2R, hY4R, and hY5R) that have been discussed in the context of several diseases [18–20]. Representing a multi-receptor/multi-ligand system, the four receptor subtypes are activated in a subtype-specific manner by three endogenous peptide ligands, namely neuropeptide Y (NPY), peptide YY (PYY), and pancreatic polypeptide (PP) [21–22]. Notably, the hY1R subtype has been discussed as promising drug target in recent years, particularly with respect to tumor diseases. Reubi and co-workers detected its pathological overexpression in ≈85% of the studied breast tumor samples and virtually all of the infiltrated lymph nodes, whereas the surrounding healthy breast tissue was found to express negligible amounts of hY1R but predominantly the closely related Y_2_ receptor subtype (hY2R) [23]. Hence, a switch from hY2R to hY1R expression during pathogenic breast-cell transformation was hypothesized. Furthermore, many breast cancers of all major breast cancer types, i.e., hormone receptor positives, HER2/neu positives, as well as triple-negatives, seem to overexpress hY1R (results not published, R. Rennert, Ontochem). Beyond breast cancers, hY1R overexpression was also detected in other cancer conditions, particularly in Ewing’s sarcoma, synovial sarcoma and leiomyosarcoma [24], but also renal cell carcinoma and nephroblastoma [25], neuroblastic tumors, paraganglioma, pheochromocytoma and adrenal cortical tumors [26], ovarian sex cord-stromal tumors and ovarian adenocarcinoma [27–28]. Besides its prevalent overexpression in tumor tissues, the NPY Y1 receptor has been identified as fast and efficiently internalizing GPCR in those cells upon agonist binding [29–30].

The NPY Y1 receptor subtype for these reasons is a very promising molecular target to be addressed by selective peptide–drug conjugates (PDCs), notably for cancer treatment or diagnosis. However, the peptide moiety of such hY1R-targeting PDCs cannot be native NPY as it is receptor-subtype unspecific. Therefore, highly hY1R-selective artificial analogues thereof are required. Consequently, a modified pig NPY analogue – namely [F^7^,P^34^]-pNPY, which is comparable to the human NPY analogue [F^7^,L^17^,P^34^]-hNPY – has been identified and claimed to be especially selective for the NPY Y1 receptor subtype in comparison to the other, very closely related NPY receptor subtypes hY2R, hY4R and hY5R [31]. Recently, Ahrens et al., in cooperation with OntoChem GmbH amongst others, tested [F^7^,P^34^]-pNPY as well as a peptide–tubulysin A conjugate [K^4^(C(TubA)-βA-),F^7^,P^34^]-pNPY – representing a comparable PDC – compared to wildtype pNPY for their binding affinities at the NPY Y1 receptor subtype. While [F^7^,P^34^]-pNPY (IC_50_ = 1.3 nM) showed a comparable binding affinity as pNPY (IC_50_ = 1.8 nM), the Y1 receptor binding of the peptide–tubulysin A conjugate [K^4^(C(TubA)-βA-),F^7^,P^34^]-pNPY (IC_50_ = 47.6 nM) was detected to be slightly reduced. However, when testing the functional receptor activation – using an second messenger (IP) accumulation assay, Ahrens and co-workers found all three peptides and PDC, respectively, in the same EC_50_ range (1.7 to 2.6 nM) at the NPY Y1 receptor. Interestingly, at the NPY Y2 receptor subtype the EC_50_ value of the subtype-unspecific wildtype pNPY was found in the same range, but the EC_50_ values of [F^7^,P^34^]-pNPY and [K^4^(C(TubA)-βA-),F^7^,P^34^]-pNPY were detected with higher than 100 nM, i.e., around two magnitudes higher than at the Y1 receptor subtype. Furthermore the authors illustrated the Y1 receptor subtype-specific endocytotic internalization of the aforementioned peptides [32]. These findings indicate the highly affine receptor binding, effective NPY Y1 receptor activation, Y1 receptor-mediated PDC internalization, as well as the payload liberation, of this type of peptide–toxin conjugate. Due to the structural identity of the used peptide moieties, we suppose a similar Y1 receptor binding and activation behavior for the tubugi-1 bearing PDC described herein, albeit not tested separately.

Meanwhile, based on this hY1R-prefering peptide [F^7^,P^34^]-pNPY, several approaches of peptide conjugates have been published with diagnostic indications [33–34]. In 2010, the Beck-Sickinger group demonstrated the suitability of hY1R-targeting for the diagnosis of NPY1R-overexpressing breast cancers in a patients pilot study (*n* = 5) by using a PET tracer based on the hY1R-specific NPY analogue [F^7^,P^34^]-pNPY [35]. This study demonstrated that it is not to be expected that NPY-based diagnostic or therapeutic PDCs will pass the blood-brain barrier and therefore could induce undesired adverse effects at the major native sites of NPY Y1 receptor occurrence and activity. Both Zwanziger et al. and Hofmann et al. later synthesized N-terminally truncated NPY analogues, namely NPY(28–36) analogues, with the intention to develop hY1R-selective agonists and conjugates of reduced size [36–37]. However, in most cases they lost more or less the hY1R binding, or selectivity, or receptor-activation efficacy, and had low metabolic stability. Besides diagnostic approaches, several therapeutic NPY-derived PDCs have been reported. Langer and co-workers conjugated daunorubicin and doxorubicin as cytotoxic drugs to native NPY by using various linker chemistries. However, due to missing hY1R-selectivity and relatively weak antitumor efficacy these conjugates were found unsuitable as PDCs [38]. More recently, further approaches of hY1R-addressing PDCs for therapeutic applications have been published, whereby the peptide moiety always is based on [F^7^,P^34^]-pNPY [32,39–40]. However, so far none of these [F^7^,P^34^]-pNPY-based conjugates proved a convincing in vivo efficacy. To further improve the general setting of peptide–drug conjugates, major efforts have been made to enhance target affinity and specificity as well as metabolic stability of the peptide moiety, and to identify novel PDC payloads permitting superior PDC efficacies.

Even with a good targeting peptide at hand, many other constrains apply to achieve a good conjugate drug: (1) the toxin (warhead, payload) must be highly active, as normal activity (medium to high nM IC_50_ like in taxanes or epothilones) [41–43] often is insufficient considering common receptor densities; (2) the linker must be designed to either not negatively affect activity of the payload, or even better to preclude activity in non-activated transport form which after recognition at the target site is cleaved to release an active form. It should be be sufficiently stable in plasma to survive delivery, and ideally should improve solubility and cell entry. After all, only very few toxins are known that are suitable for PDCs, and the design and synthesis of suitable linkers is a task of crucial importance and synthetic challenge that still is underestimated by many entering the field.

**Figure 1:**
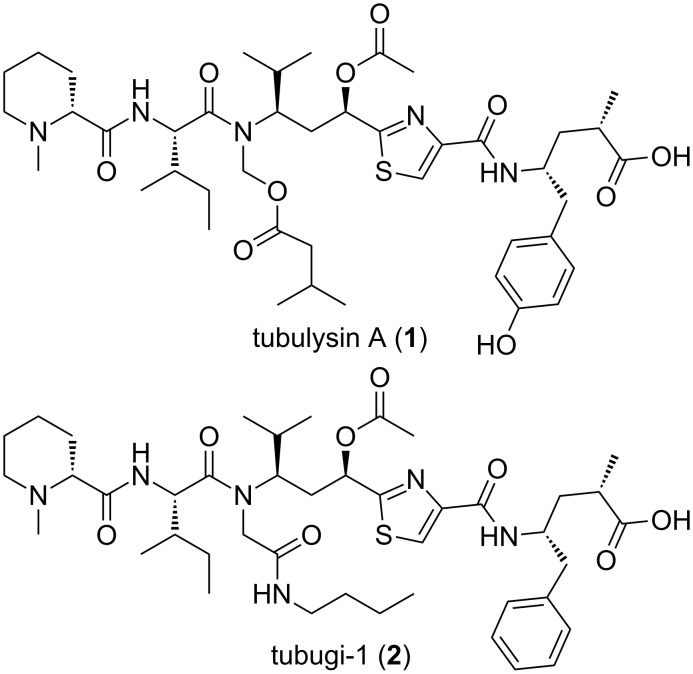
Tubulysin A (**1**) and tubugi-1 (**2**).

The most promising PDC payloads, often also referred to as ‘warheads’, are toxins of limited molecular size but with outstanding potency in the picomolar or lower concentration range. Consequently, the few candidates often have a very narrow or even non existing therapeutic window as stand-alone drug. Recently, our group was the first to publish total synthetic strategies towards tubulysins and the so-called tubugis, the latter as more suitable 2nd generation derivatives (Figure 1) [44–46]. Tubulysins were originally discovered and isolated from myxobacteria [47–48], with picomolar in vitro activities [45–46,49–54], that are caused by a destabilization and degradation of the microtubuli network undermining its function in mitosis of eukaryotic cells. Hence, these toxins primarily affect fast dividing cells, for instance all active cancer cells. Tubugis as derivatives of natural tubulysins have an almost identical antitumor activity, but are readily available and, most importantly, are chemically more inert and less degradable than native tubulysins.

The aim of this work was to prepare a novel branched NPY Y1-receptor-selective peptide–toxin conjugate version with a tubugi toxin. Therefore, the NPY analogue [K^4^(C-βA-),F^7^,L^17^,P^34^]-hNPY was conjugated with tubugi-1 (**2**) as therapeutic payload, using a linker that promises a more general use than just for the present case. The study furthermore comprises the investigation of the PDCs’ in vitro efficacies on the viability and proliferation of colon and prostate as well as several breast cancer and Ewing`s sarcoma cell lines. Thereby the correlation with the hY1R expression levels of the latter three cell lines was determined as proof for targeted delivery.

## Results and Discussion

### Synthesis of tubugi-1 building blocks

We established the Ugi reaction as a powerful tool for peptide synthesis and ligation, including the first syntheses of tubulysine derivatives by us and later also others [44,53,55–56]. In a single step, the Ugi reaction permits the introduction of different functionalities which may be followed with additional modifications on the side chain (e.g., via ring-closing metathesis or Click reaction) [53].

For tubugi conjugates we learned that alkyl amide bonds and several types of linkers are unsuitable, as they rendered the peptide inactive (results not shown). However, disulfide-bonded linkers retained activity, presumably by cleavage in the reductive milieu of cancer cells, if connected via a short ester or amide linkage at the C-terminus.

**Scheme 1:**
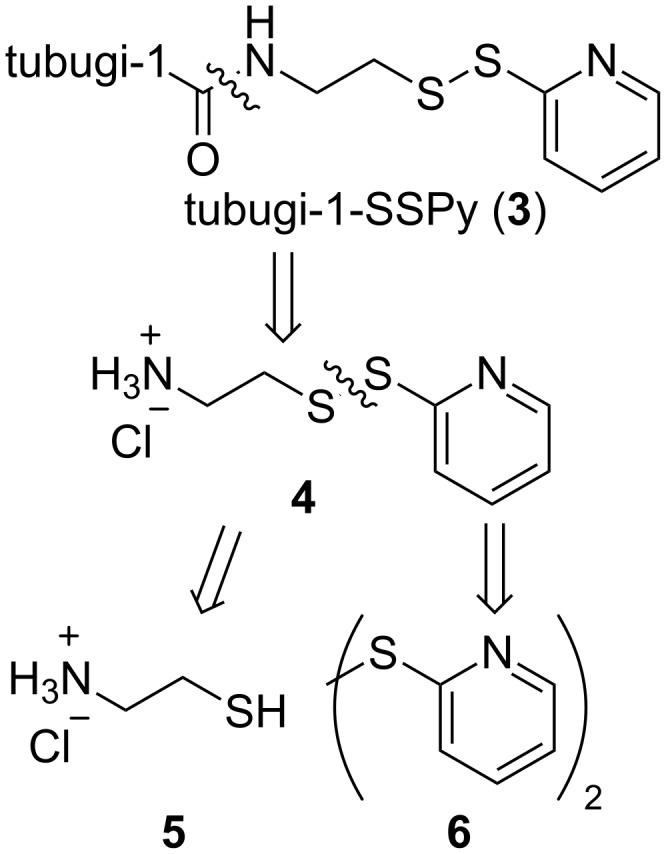
Retrosynthetic analysis of the modular attachment linker tubugi-1-SSPy (**3**).

The retrosynthetic analysis (Scheme 1) shows that, in addition to tubugi-1 itself, only the readily accessible building block **4** is required to construct the activated compound tubugi-1-SSPy (**3**) as a universal precursor for peptide–toxin conjugate syntheses [57]. The pyridyl disulfide is a leaving group which can be substituted by all nucleophilic thiolates (bound to various target peptides) by directed disulfide exchange. Compound **4** is accessible by reaction of the commercially available substances cysteamine (**5**) and 2,2'-dithiodipyridine (**6**).

**Scheme 2:**
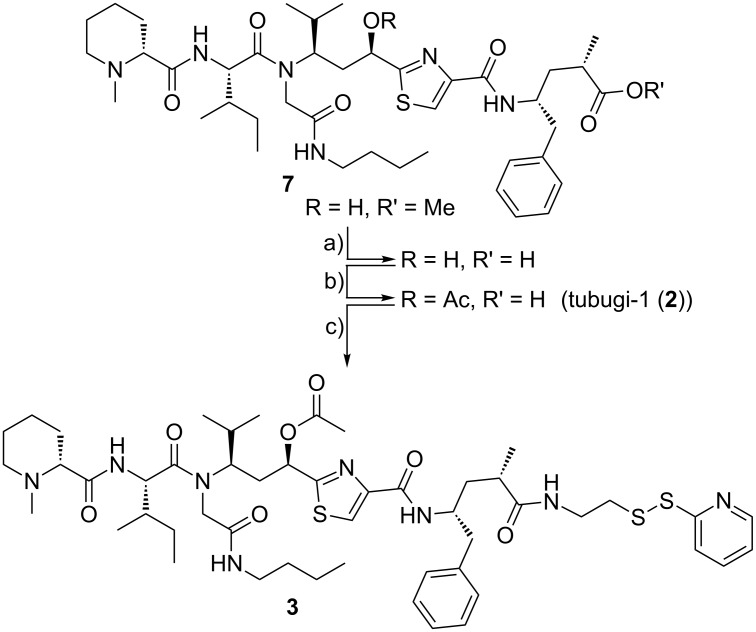
Synthesis of tubugi-1-SSPy (**3**): a) LiOH·H_2_O, THF/H_2_O, 0 °C → rt; b) Ac_2_O, py; c) **4**, HBTU, DMF, DIPEA, MeOH, under N_2_ atmosphere, 42% (3 steps).

In practice, the synthesis of tubugi-1-SSPy from the published methyl ester precursor **7** is more efficient via the non-acetylated tubugi-1, because in this case it is not necessary to isolate tubugi-1 (**2**) itself (Scheme 2). Therefore, methyl ester **7** is hydrolyzed at all ester bonds, and the resulting acid is acetylated at the tubuvaline hydroxy group to give tubugi-1 (**2**). Without isolation this is directly converted using building block **4** and HBTU and DIPEA as reagents to give tubugi-1-SSPy (**3**, Scheme 2). Purification by column chromatography finally yields the target compound tubugi-1-SSPy (**3**), which constitutes the payload with a rather universally pre-activated linker.

The disulfide linkage was chosen for the tubugi-1 coupling to the peptide moiety due to own promising preliminary work. Several linker chemistries were tested with tubulysin-like peptides – amongst them amide and ester linkers, hydrazone linker, VC linker etc. – the disulfide linker described herein, however, showed the best performance regarding synthetic practicability in conjunction with tubugi-1 and a peptide moiety, as well as the best results liberating the toxin from the conjugate.

### Synthesis of hY1R-targeting PDC using tubugi-1 (**2**)

**Scheme 3:**
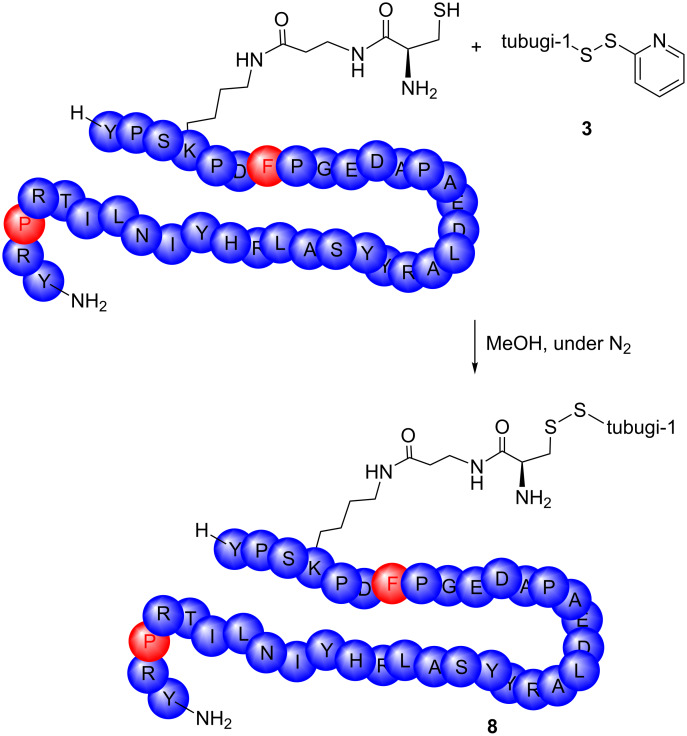
Synthesis of the tubugi-1–NPY conjugate [K^4^(C(tubugi-1)-βA-),F^7^,L^17^,P^34^]-hNPY (**8**).

The peptide–toxin conjugate bearing the payload tubugi-1, [K^4^(C(tubugi-1)-βA),F^7^,L^17^,P^34^]-hNPY (**8**), was synthesized by reacting the tubugi-1-SSPy (**3**) with the free thiol function of a β-alanine–cysteine dipeptide (βAC) linked to the side chain of Lys^4^ of the targeting peptide. For this purpose, 1 mol equiv of the tubugi-1-SSPy building block **3** and one molar equivalent of the targeting peptide, [K^4^(C-βA-),F^7^,L^17^,P^34^]-hNPY, were reacted for 60 min in an air- and moisture-free atmosphere (Scheme 3). The desired tubugi-1–NPY conjugate **8** with cleavable disulfide bridge was isolated by RP-HPLC and the purity of the substance was determined by analytical HPLC. The conjugate **8** was characterized by ESI–FTICR–MS measurements (see Supporting Information File 1). All signals for [M + *n*H]*^n^*^+^ with *n* = 4–8 could be identified.

**Scheme 4:**
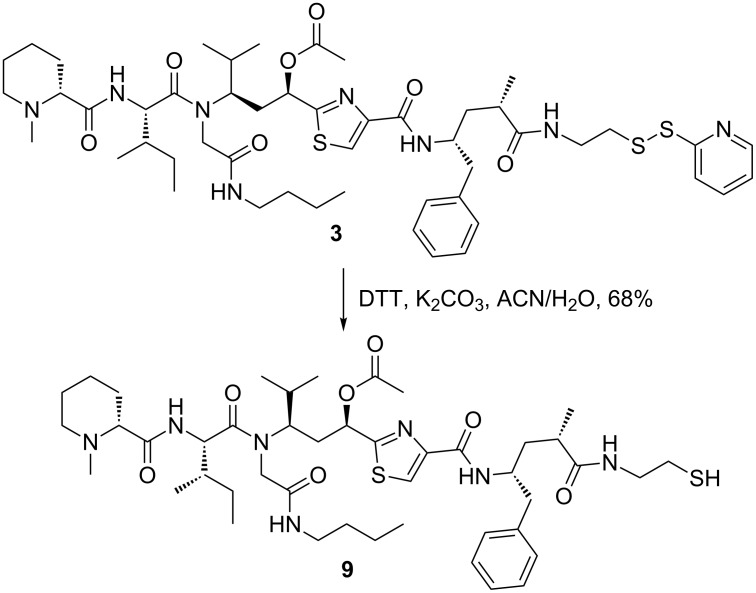
Toxin liberation by disulfide linker cleavage from the activated toxin conjugate under reductive conditions using DTT.

After NPY Y_1_ receptor-mediated, endocytotic accumulation of the respective peptide–toxin conjugate **8** in the targeted tumor cells, the cytotoxic tubugi-1 should be released by cleavage of the disulfide bridge in the highly reducing environment of the endo-lysosomal compartments. From the synthetic point of view, this compound is accessible, as shown by reduction of tubugi-1-SSPy (**3**) with DTT (Scheme 4). Comparable reactivity is expected within the endo-lysosomal compartments after NPY Y_1_ receptor-mediated internalization of the peptide–toxin conjugate into the target cells via clathrin-dependent endocytosis. In the following, in vitro studies were conducted to verify if this expectation is met, and to study the biological consequences thereof with respect to the antitumor impact of the peptide–toxin conjugate **8**.

### Effect on cell viability and proliferation

Due to their very high toxic potency, tubulysins as well as their synthetic tubugi analogues can also exhibit toxic effects on healthy cells. Therefore, considerable adverse effects can occur in vivo in case of untargeted applications. For that reason, feasible therapeutic windows of this class of toxins are only realistic if the toxins are applied as ‘detoxified’ prodrugs, e.g., in the form of peptide–toxin conjugates, whereby the effect of tubulysin or tubugi, respectively, is strongly hampered in its cytotoxic activity, and the peptide moiety ensures the target-specific toxin delivery toward the diseased cells, while omitting (most) healthy cells.

**Figure 2:**
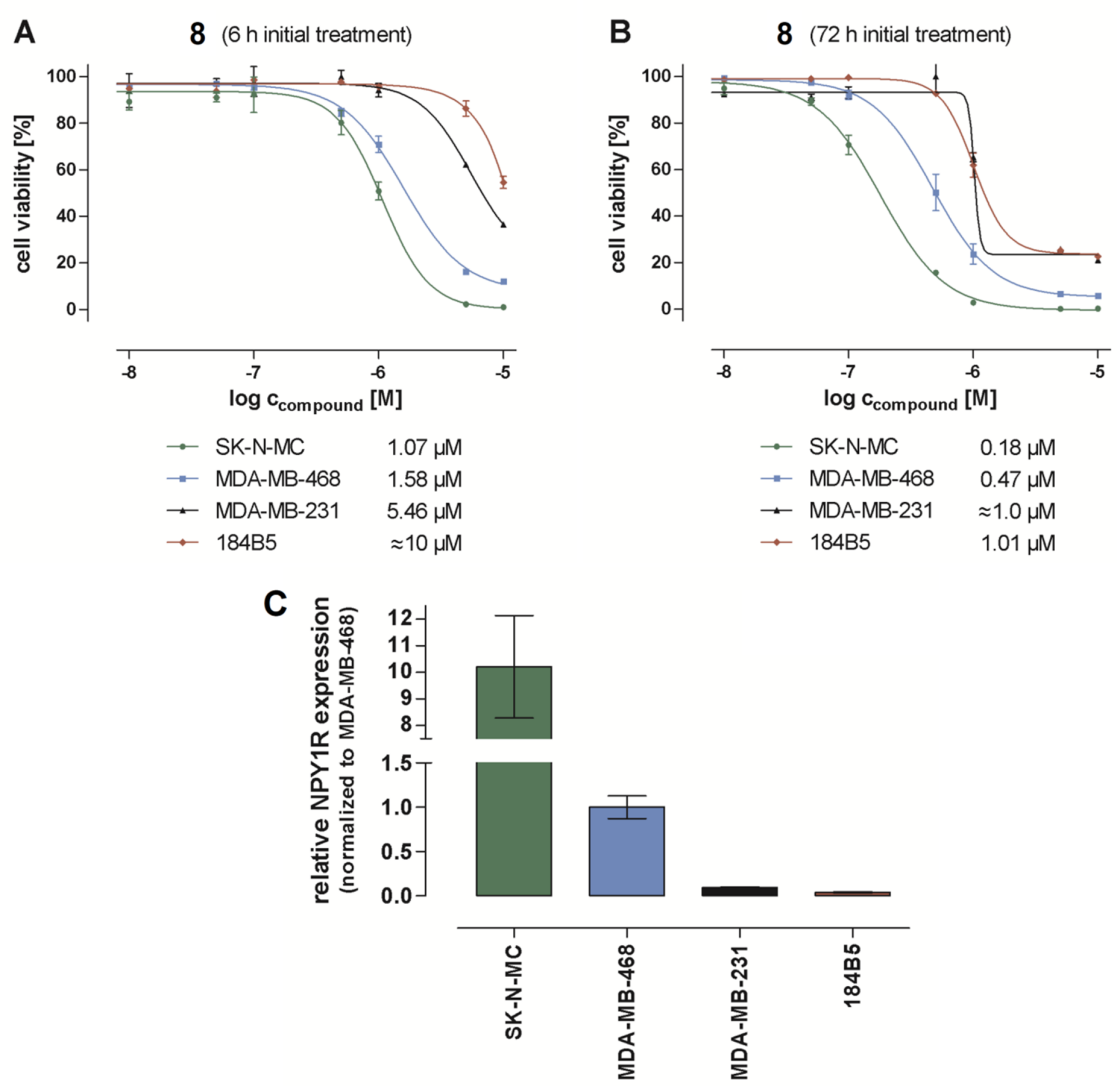
Reduction of viability and proliferation of SK-N-MC, MDA-MB-468, MDA-MB-231 cancer cell lines, and normal mammary gland epithelium cell line 184B5 treated with NPY Y1 receptor-targeting peptide–toxin conjugate **8**: (A) pulse setting (initial 6 h treatment followed by 66 h growth in PDC-free standard medium); (B) 72 h treatment; (C) NPY Y1 receptor expression of the SK-N-MC, MDA-MB-468, MDA-MB-231 and 184B5 cell lines determined by using RT-qPCR quantification; the NPY1R expression levels are normalized to NPY1R expression level in MDA-MB-468 cells (set to 1.0).

To assess the impact of the chemical modifications due to the linker-assisted peptide–toxin conjugation and toxin liberation on the tubugi-1 toxin’s, in vitro antitumor efficiency of free tubugi-1 (**2**), tubugi-1–NPY-derived conjugate **8**, as well as its reduced linker product **9** were initially tested against HT-29, PC-3 and Colo320 tumor cells. Contrarily to, for instance, SK-N-MC cells shown in Figure 2, the three aforementioned cell lines are not known for high NPY receptor expression levels. Throughout the three cell lines, PC-3 expresses the highest level of NPY Y1 receptor [58], but by magnitudes lower than SK-N-MC for instance. This might also explain the gap of the toxic potencies (factor ≈1.000) of **2** and **8**, respectively, as shown in Table 1. Furthermore, the PC-3 cells were indeed detected to be the most sensitive cell line compared to HT-29 and Colo320, since PC-3 probably expresses a higher NPY Y1 receptor level and internalizes, consequently, more peptide–toxin conjugate.

**Table 1:** IC_50_ values [nM] of the reference and linker-modified toxin against HT-29, PC-3 and Colo320 cell lines.

compound		IC_50_ [nM]		
		
		HT-29	PC-3	Colo320

**1**	tubulysin A	0.21 ± 0.05	0.32 ± 0.06	0.38 ± 0.01
**2**	tubugi-1	0.14 ± 0.02	0.23 ± 0.05	0.46 ± 0.05
**8**	tubugi-1–SS–NPY	452 ± 60	205 ± 49	706 ± 185
**9**	tubugi-1-SH	60 ± 6	41 ± 8	556 ± 77

The antiproliferative activities of the investigated compounds and conjugates are summarized in Table 1. Natural tubulysin A (**1**), used for comparison, and the synthetic analogue tubugi-1 (**2**) expressed similar cytotoxic activities against the selected cancer cell lines in medium pM concentrations. Both compounds, **1** and **2**, are able to penetrate the cells’ membrane by unspecific, receptor-independent pathways, not discriminating between normal and transformed cells. For that reason it is very difficult to adjust a practicable therapeutic window for these toxins. All the more, it is important to mask the high toxicity of tubulysin A and tubugi-1 until the toxins are delivered to the cells targeted. Indeed, in its conjugated form, represented by **8**, attached to the peptide moiety designed to target the NPY Y1 receptor, the toxicity of tubugi-1 was found to be masked, with IC_50_ values increased ≈1,000-fold compared to the free toxin. The restoration of the tubugi-1 toxicity presupposes the intracellular cleavage of the disulfide linker within the reducing environment of the endo-lysosomal compartments of the addressed tumor cells, what should be simulated by testing tubugi-1-SH (**9**). As shown in Table 1, the cytotoxic potency of the tubugi-1-SH was – in case of HT-29 and PC-3 – by factors ≈5 to 8 higher compared to the entire peptide–toxin conjugate **8**. The only slight increase of cytotoxic activity of compound **9** compared to the complete conjugate **8** in Colo320 cells is most likely caused by a generally weak responsiveness of Colo320 cells towards tubugi-1-SH and the entire conjugate tubugi-1–SS–NPY. When compared with HT-29 and PC-3 cells, the IC50 value of tubugi-1-SH is by factor 10 higher in Colo320. Since the membrane passage of tubugi-1-SH is not depending on a NPY receptor, there have to be other explanations for the reduced cytotoxic impact of tubugi-1 and corresponding derivatives in Colo320, rather than the NPY Y1 receptor expression level.

A significant aspect of the present concept of a hY1R-targeting peptide–toxin conjugate is the fact that intact tubugi-1–NPY conjugate **8** permits in the systemic situation before reaching the target cells much lower toxicity than the cytotoxic compound tubugi-1 alone, thus opening a feasible therapeutic window for the class of tubugi toxins. In that context, a loss of tubugi-1 activity is expectable due to its chemical modification caused by the linker-based conjugation, and after linker cleavage the intracellular activities of **8**, i.e., the activities of the linker cleavage product **9**, are within an acceptable range, and are comparable or higher than that of some commercially used anticancer compounds (e.g., cisplatin and doxorubicin). Further in vitro cell proliferation and viability assays were conducted to investigate the impact of various durations of incubation of **8**, and for the correlation of its potency with the hY1R expression levels of the cells.

For that reason, a collection of tumor cell lines was used that represents a wide range of cellular hY1R expression levels, i.e., highly hY1R-overexpressing Ewing`s sarcoma SK-N-MC cells, the triple-negative breast cancer cell lines MDA-MB-468 and MDA-MB-321 which are moderately and weakly expressing, respectively, as well as the 184B5 cell line, representing a normal, but chemically immortalized mammary gland epithelium with very weak hY1R expression. These cell lines were incubated with PDC **8** in two treatment regimens. One regime considered a pulsed setting, i.e., initial treatment with the drug for 6 h, washing and subsequently culturing without the PDC to reach 72 h (Figure 2A). In the second regime, the cells were treated for the whole 72 h period with the PDC (Figure 2B). As to be expected, the 72 h treatment is more effective than the 6 h pulse treatment. Notably, in vitro antitumor activities of **8** were found to correlate very good with the hY1R expression levels, as detected by gene expression analyses using RT-qPCR (Figure 2C). Both the cytotoxic activity and the hY1R expression level rank in the order SK-N-MC > MDA-MB-468 > MDA-MB-231 > 184B5, what proofs the hY1R-specific and -selective nature of the mode of antitumor action of the designed PDC **8**. Importantly, the activity of **8** against the selected normal breast cell line 184B5 is in the same order of magnitude as for the hY1R-deficient tumor cell line (MDA-MB-231), both tested at even higher concentration of the PDC than for the Y1 cell lines. This points out good selectivity not only between tumor cell lines with the different hY1R expression levels but also good discrimination against normal (non-cancerous) cells.

## Conclusion

The highly active cytotoxin tubugi-1 was successfully conjugated to a truncated and modified neuropeptide-Y mimetic to form a new peptide–toxin conjugate (PDC **8**) with a reductively cleavable disulfide linker. The tubugi-1–NPY conjugate has a strongly masked antitumor activity against HT-29, PC-3 and Colo320 cells in comparison to the active compound alone, but the activity is restored to a sufficient extent upon linker cleavage (tubugi-1–SS–NPY → tubugi-1-SH). Most importantly, the cytotoxic potential of tubugi-1–SS–NPY correlates very well with the hY1R expression levels of a panel of tumor cell lines. For instance, the hY1R-overexpressing Ewing`s sarcoma cell line SK-N-MC was much more affected by the PDC than the normal (but chemically transformed) cell line 184B5 with weak hY1R expression. However, further efforts should be made to improve activity after internalization of the PDC.

Overall, the investigations carried out up to this point provide a biological validation of the developed conjugate. The principally modular conjugation protocol for tubugis bears promise for further cancer targeting conjugates.

## Supporting Information

File 1 Complete experimental procedures and characterization data.
