# Supplementary material for: Synthesis of a tubugi-1-toxin conjugate by a modulizable disulfide linker system with a neuropeptide Y analogue showing selectivity for hY1R-overexpressing tumor cells
Source: Beilstein J Org Chem. 2019 Jan 10;15:96–105. doi: 10.3762/bjoc.15.11 (PMC6334802; doi:10.3762/bjoc.15.11)
Supplement: File 1 — Complete experimental procedures and characterization data. [file Beilstein_J_Org_Chem-15-96-s001.pdf]

BEILSTEIN JOURNAL OF ORGANIC CHEMISTRY

Supporting Information

for

Synthesis of a tubugi-1-toxin conjugate by a modulizable disulfide linker system with a neuropeptide Y analogue showing selectivity for hY1R-overexpressing tumor cells

Rainer Kufka, Robert Rennert, Goran N. Kaluđerović, Lutz Weber, Wolfgang Richter and Ludger A. Wessjohann

Beilstein J. Org. Chem. **2019,** *15,* 96–105. doi:10.3762/bjoc.15.11

Complete experimental procedures and characterization data

License and Terms: This is a supporting information file under the terms of the Creative Commons Attribution License (http://creativecommons.org/licenses/by/4.0). Please note that the reuse, redistribution and reproduction in particular requires that the authors and source are credited.
The license is subject to the *Beilstein Journal of Organic Chemistry* terms and conditions: (https://www.beilstein-journals.org/bjoc)

Experimental part

General

All commercially available chemicals were used without further purification. ^{1}H NMR ($CDCl_3$, 400 MHz) and ^{13}C NMR ($CDCl_3$, 100 MHz) spectra were recorded in $CDCl_3$ solutions on a Varian Mercury 400 spectrometer at 400 (^{1}H) and 100 MHz (^{13}C), respectively. Chemical shifts (δ) are reported in ppm relative to TMS (^{1}H-NMR) and to residual $CDCl_3$ signal (^{13}C-NMR). High resolution ESI mass spectra were obtained from a Bruker Apex III Fourier transform ion cyclotron resonance (FT-ICR) mass spectrometer equipped with an Infinity™ cell, a 7.0 Tesla superconducting magnet, an RF-only hexapole ion guide and an external electrospray ion source (Agilent, off axis spray). ESI-MS was recorded on a Finnigan TSQ 7000, LC-Tech Ultra Plus pumps, Linear UV-VIS 200 detector, Sepserve Ultrasep ES RP-18 5 μm 1×100 mm column, flow 70 $\mu L \cdot min^{-1}$. For mass analysis using MALDI-TOF mass spectrometry a Bruker Daltonics Ultraflex III TOF/TOF instrumentation was used, whereby the matrix consisted of 2,5-dihydroxybenzoic acid and 2-hydroxy-5-methoxybenzoic acid (10 g/L in ACN/H_2O/TFA 50 : 49.7 : 0.3 v/v). Alternatively, MALDI-TOF analyses were conducted by using a Bruker Autoflex Speed TOF/TOF system (Bruker) with a 2,5-dihydroxyacetophenone matrix.

Flash column chromatography was carried out using Merck silica gel 60 (0.040-0.063 mm) and analytical thin layer (TLC) was performed using Merck silica gel 60 F254 aluminium sheets. HPLC experiments were performed in an Agilent 1100 series equipped with a column SNr. 176: YMC pack 150 x 4.6 LD 102 Å 5 μm ODS-A and UV detector (200-600 nm). Tubulysin A (**1**) was obtained commercially and

used without purification (TUBE Pharmaceuticals GmbH, Vienna, Austria). Tubugi-1 precursor **7**, the non-acetylated methyl ester, was synthesized by the method published previously [1].

Preparation of *S*-(2-pyridylthio)cysteamine hydrochloride (4)

2.2 ''-Dithiopyridine (**6**, 1.1 g, 5.0 mmol, 1.1 eq.) is added dropwise to a solution of cysteamine hydrochloride (**5**, 516 mg, 4.5 mmol, 1.0 eq.) in MeOH (10 mL). The reaction mixture is stirred for 90 minutes and the solvent is removed at 30 °C. under reduced pressure. Column column chromatography (DCM: MeOH 10:1) of the residue provides compound **4** as a white solid.

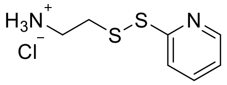

Yield: 439 mg (49%). Purified by column chromatography. R_f 0.18 (DCM/MeOH 10:1). ^{1}H NMR (CD_3OD, 400 MHz): δ (ppm) 8.51–8.48 (m, 1H), 7.82–7.77 (m, 1H), 7.71–7.68 (m, 1H), 7.31–7.26 (m, 1H), 3.19 (d, *J* = 6.0 Hz, 2H), 3.06 (d, *J* = 6.0 Hz, 2H). HRMS (ESI-pos) m/z calcd for $C_7H_{11}N_2S_2$ $(M)^+$ 187.0358, found 187.0356.

Peptide Synthesis

Peptides were synthesized according to the Fmoc/*t*-Bu strategy using an automated multiple solid-phase peptide synthesizer Syro II (MultiSynTech GmbH, Bochum, Germany). For the synthesis of C-terminal peptide amides, a Rink amide resin with a loading capacity of 0.63 mmol/g was used.

Prior to each amino acid coupling step the base-labile N^{α}-protecting group Fmoc had to be cleaved off from the building blocks, and in a first step from the Rink amide resin as well. For Fmoc cleavage, 400 µL piperidine in DMF (40% v/v) was added to the resin and incubated for 3 min while stirring. The deprotection was repeated with 400 µL piperidine in DMF (20% v/v) for 10 min. Subsequently, the resin was washed with 4x 600 µL DMF.

Amino acids were coupled by preincubation of the resin with 200 µL amino acid building block solution (0.5 M in DMF) and 100 µL 3 M Oxyma in DMF for 2 min. Subsequently, 100 µL 3.3 M DIC in DMF were added and the reaction was allowed to proceed for 40 min with stirring. After a washing step with 800 µL DMF, the coupling step was repeated once for each amino acid.

For the synthesis of NPY analogues with sequence branching at position Lys^4 a synthetic method previously described by Ahrens and coworkers was used [2]. In brief, the peptide sequence was built up to yield Dde-[K^4(Fmoc),F^7,L^{17},P^{34}]-hNPY(4-36) with Lys^4 terminally protected with Dde protecting at N^{α} and Fmoc protecting group at N^{ε}. In the next step Fmoc of N^{ε} of Lys^4 was cleaved off and the sequence was elongated at this terminus by sequential introduction of β-alanine and cysteine. Subsequently, the *N*-terminal Dde group of Lys^4 was selectively removed from the resin-bound peptide. For that purpose the resin was incubated 12x 10 min with 1 mL freshly prepared 3% hydrazine in DMF. After each of the 12 steps, the resin was washed with DMF. After the first and the twelfth step, the removed hydrazine solution was collected, and its absorption was measured at 301 nm against a reference of fresh hydrazine in DMF. The Dde deprotection was successful if the absorption of

the final (10th) fraction was < 0.1. The recruited Lys^4 *N*-terminus was then elongated by standard coupling steps to yield the branched full-length peptide [K^4(C-βA-),F^7,L^{17},P^{34}]-hNPY(1-36). Purification of the synthesized [K^4(C-βA-),F^7,L^{17},P^{34}]-hNPY peptide was achieved by preparative RP-HPLC on a Phenomenex Jupiter Proteo C18 column (21.2 mm x 250 mm) using an elution system composed of (A) 0.1% TFA in H_2O and (B) 0.08% TFA in ACN, and an appropriate linear gradient of solvent B in A over 40 – 50 min and a flow rate of 10 $mL \cdot min^{-1}$. For peptide detection, absorption at 220 nm was measured. Fractions were analyzed by MALDI-TOF mass spectrometry and analytical RP-HPLC. Those fractions identified to contain the pure product were combined and lyophilized.

Preparation of tubugi-1-SSPy (3)

LiOH (21.0 mg, 0.5 mmol, 5.0 eq.) was added at 0 °C to tubugi-1 precursor compound **7** (80 mg, 0.1 mmol, 1.0 eq.), dissolved in THF/H_2O (1 mL / 0.5 mL). The reaction mixture was stirred overnight at room temperature and then concentrated to dryness under reduced pressure. The residue was dissolved in H_2O (2.5 mL), pH was adjusted to **2** with saturated $NaHSO_4$ solution and extracted with EtOAc (5× 2 mL). The combined organic phases were dried over Na_2SO_4 sodium sulfate and filtered. The solvent was removed under reduced pressure. Acetic anhydride (75 µL, 0.8 mmol, 8 eq.) was added at 0 °C to a solution of the resulting residue in dry pyridine (1 mL). The reaction mixture was stirred overnight at room temperature and then cooled to 0 °C. A mixture of dioxane/H_2O (1 mL / 1 mL) was added and the mixture was stirred overnight, and afterwards concentrated to dryness under reduced pressure. The crude tubugi-1 (**2**), used without further purification, and

HBTU (41.7 mg, 0.11 mmol, 1.1 eq.) were dissolved in DMF (4 mL) and added dropwise at 0 °C under a nitrogen atmosphere to a solution of compound **4** (42.8 mg, 0.2 mmol, 2.0 eq) and DIPEA (94.0 µL, 0.55 mmol, 5.5 eq.) in dry MeOH (4 mL). The reaction mixture was stirred for 2 h and then concentrated to dryness under reduced pressure. Column chromatographic purification (DCM: MeOH 10:1 → 50:1) of the residue yielded 41 mg (42% over 3 steps) tubugi-1-SSPy (**3**) as a white solid.

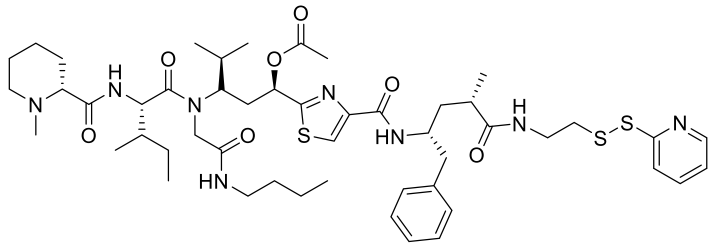

3: Rf = 0.48 (DCM:MeOH 10:1). ^{1}H NMR (CD_3OD, 400 MHz): δ (ppm) 8.39 – 8.36 (m, 1H), 8.04 (s, 1H), 7.84 – 7.75 (m, 3H), 7.20 – 7.18 (m, 5H), 6.35 (d, *J* = 9.8, 1H), 4.79 – 4.73 (m, 1H), 4.64 – 4.55 (m, 2H), 4.45 – 4.38 (m, 1H), 4.32 – 4.24 (m, 1H), 3.80 – 3.72 (m, 1H), 3.58 – 3.49 (m, 1H), 3.44 – 3.36 (m, 1H), 3.25 (d, *J*=7.0, 1H), 3.18 – 3.06 (m, 2H), 2.97 – 2.90 (m, 3H), 2.85 (dd, *J* = 11.3, 7.0, 2H), 2.59 – 2.52 (m, 1H), 2.49 – 2.42 (m, 1H), 2.32 – 2.24 (m, 1H), 1.80 – 1.71 (m, 3H), 1.66 – 1.53 (m, 6H), 1.48 – 1.41 (m, 2H), 1.33 – 1.27 (m, 5H), 1.12 – 1.04 (m, 8H), 0.97 (d, *J* = 6.7, 4H), 0.91 – 0.82 (m, 9H), 0.76 ppm (t, *J* = 5.9, 3H). ^{13}C NMR (CD_3OD, 101 MHz): δ (ppm) 178.68, 175.79, 172.08, 171.83, 171.17, 163.18, 161.20, 150.56, 150.41, 139.53, 139.12, 130.42, 129.31, 127.36, 125.28, 122.90, 122.38, 121.32, 71.41, 70.35, 56.67, 54.94, 50.79, 44.73, 44.58, 42.63, 40.54, 39.86, 39.29, 39.05, 38.96, 37.20, 36.66, 32.60, 31.63, 31.13, 26.20, 25.65, 24.33, 21.11, 20.94, 20.90, 20.12, 19.10, 16.37, 14.12, 10.69. HRMS (ESI-pos) *m/z* calcd for $C_{50}H_{74}N_8O_7S_3$ $[M+2H]^{2+}$ 498.2494, found 498.2493.

Toxin coupling to the peptide moiety by disulfide exchange

For the synthesis of the peptide-toxin conjugate **8**, equimolar quantities of the peptide (dissolved in degassed PBS, pH 6.0, with 20% ACN, v/v) and the linker-equipped toxin (**3**; dissolved in DMSO) were mixed 1:1 (v/v) and reacted under nitrogen atmosphere at room temperature for 60 min. Purification was performed using RP-HPLC (150 mm × 4.6 mm YMC ODS-A; Figure S1). The elution system was composed of (A) 0.1% TFA in ACN and (B) 0.1% TFA in H_2O. A linear gradient of 60 – 30% solvent A in B over 30 min with a flow rate of 14 $mL \cdot min^{-1}$ was used. The conjugate **8** was detected at 220 nm and subsequently analyzed by ESI-FTICR mass spectrometry (Table S1).

Table S1: ESI-FTICR-MS of tubugi-1-NPY conjugate (**8**)

m/*z* (measured)	*m*/*z* (calculated)	Error (ppm)	$[M + nH]^{n+}$	A ($m/z_{(measured)} \times n$)	$A–nH^+$
664.8340	664.8399	0.1	$[M + 8H]^{8+}$	5318.6720	5310.6136
759.6726	759.6731	0.7	$[M + 7H]^{7+}$	5317.7082	5310.6571
886.1181	886.1174	0.8	$[M + 6H]^{6+}$	5316.7086	5310.6648
1063.1411	1063.1395	1.5	$[M + 5H]^{5+}$	5315.7055	5310.6690
1328.6740	1328.6725	1.1	$[M + 4H]^{4+}$	5314.7096	5310.6804

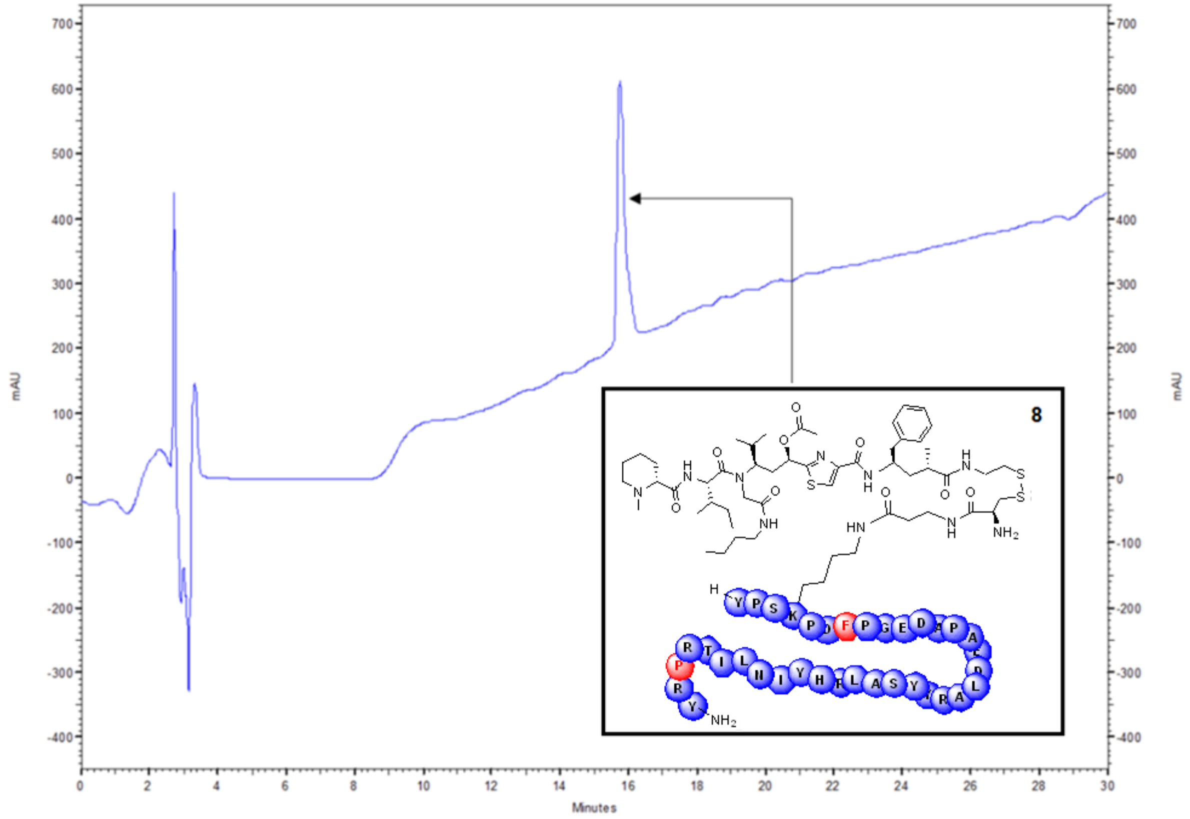

Figure S1: HPL chromatogram of tubugi-1-NPY conjugate (**8**). Please note that the NPY-mimetic part shows the peptide inverse to standard direction (N-terminus at right/top, C-terminus as amide at left/bottom) for the sake of saving space.

Cleavage of tubugi-1-SSPy (3→9)

K_2CO_3 (0.2 M, 50 µL) and dithiothreitol (DTT, 1.0 mg, 6.0 µmol, 1.5 eq.) were added to a solution of **3** (3.9 mg, 3.9 µmol, 1 eq.) in acetonitrile/H_2O (150 µL/75 µL). The reaction mixture was stirred for 25 min, subsequently the solvent was removed in a rotavap. Column chromatography (CH_2Cl_2:MeOH = 40:1) of the crude product yielded 2.4 mg (68%) of **9**.

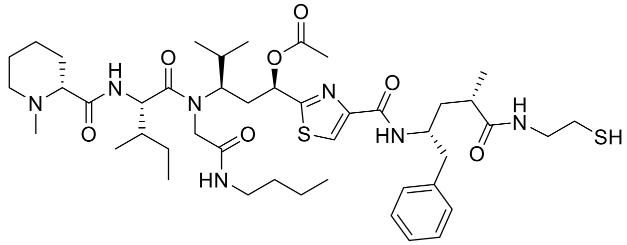

9: Rf = 0.64 (DCM:MeOH 10:1). ^{1}H NMR (CD_3OD, 600 MHz): δ (ppm) 8.06 (s, 1H), 7.23 – 7.18 (m, 4H), 7.16 – 7.10 (m, 1H), 6.35 (d, J=11.6, 1H), 4.63 – 4.56 (m, 1H), 4.45 – 4.38 (m, 1H), 4.31 – 4.25 (m, 1H), 3.82 – 3.74 (m, 1H), 3.63 (s, 1H), 3.51 – 3.45 (m, 6H), 3.05 – 3.00 (m, 4H), 2.90–2.81 (m, 9H), 2.67 – 2.57 (m, 2H), 2.24 – 2.20 (m, 1H), 2.16 – 2.14 (m, 1H), 1.64–1.52 (m, 5H), 1.34 – 1.28 (m, 5H), 1.10 – 1.05 (m, 6H), 0.99 – 0.96 (m, 3H), 0.93 – 0.84 (m, 9H), 0.80 – 0.73 ppm (m, 3H). ^{13}C NMR (CD_3OD, 151 MHz,): δ (ppm) 178.69, 175.73, 172.10, 171.76, 171.13, 163.19, 150.56, 139.54, 134.47, 130.42, 129.91, 129.48, 129.31, 127.38, 114.94, 71.57, 56.64, 54.99, 50.83, 50.39, 44.47, 44.43, 43.94, 42.60, 41.79, 40.57, 32.61, 31.81, 31.12, 30.77, 24.55, 24.20, 21.11, 20.89, 20.09, 19.17, 14.12 ppm. HRMS (ESI-pos) *m/z* calcd for $C_{45}H_{71}N_7O_7S_2$ $[M+H]^+$ 886.4929, found 886.4926.

Biological assays

Cell Culture

The colon cancer cell lines HT-29 and Colo320, the prostate cancer cell line PC-3, breast cancer cell lines MDA-MB-231 and MDA-MB-468, the Ewing`s sarcoma cell line SK-N-MC, as well as the chemically transformed normal mammary gland epithelium cell line 184B5 were cultured in a humidified atmosphere with 5% CO_2 at 37°C, and routinely grown to subconfluency (~ 70%) prior to use or subculturing. HT-

29, Colo320 and PC-3 cells were grown in RPMI 1640 completed medium (RPMI 1640 medium with 2 mM L-glutamine and 10% heat-inactivated FCS). MDA-MB-231 and MDA-MB-468 cells were grown in completed medium supplemented with 0.3 U/mL (10 µg/mL) insulin. SK-N-MC cells were cultured in DMEM (high glucose, i.e. 4.5 g/L) supplemented with 10% (v/v) heat-inactivated FCS, 2 mM L-glutamine, 0.2% NEAA and 1 mM sodium pyruvate. 184B5 cells were cultured in Pantum 468 medium supplemented with 10% (v/v) heat-inactivated FCS, 2 mM L-glutamine and 0.8% (v/v) G5 supplement.

All basal media, PBS, L-glutamine, NEAA, sodium pyruvate and trypsin/EDTA for cell culturing were purchased from PAA Laboratories (Cölbe, Germany) and PAN-Biotech (Aidenbach, Germany), respectively. FCS was from PAA Laboratories (FCS Gold) and Biochrom (Berlin, Germany; FBS Superior), respectively. G5 supplement was purchased from PAA Laboratories and Life Technologies GmbH (Darmstadt, Germany), respectively. Human insulin (recomb., expressed in yeast) was purchased from Sigma-Aldrich (Taufkirchen, Germany). Culture flasks and plates were used from TPP (Trasadingen, Switzerland), further cell culture plastics were from TPP and Corning (Wiesbaden, Germany), respectively.

Cell Proliferation Assay (*in vitro*)

Antiproliferative and cytotoxic effects, respectively, of the compounds were detected by using a fluorometric resazurin-based cell viability assay (*in vitro* toxicology assay kit; Sigma-Aldrich, Taufkirchen, Germany). Colon (HT-29 and Colo320), prostate (PC-3), breast cancer cell lines (MDA-MB-468 and MDA-MB-231), the chemically transformed but normal mammary gland epithelium cell line 184B5, as well as the

Ewing`s sarcoma family cell line SK-N-MC were seeded with low densities into 96-well plates (4,000 – 20,000 cells per well; seeding confluency ~ 10 – 20%), and were allowed to adhere for 24 h. Subsequently, the compounds and peptide-toxin conjugate **8** – diluted to appropriate concentrations in the respective culture medium – were added to the cells. HT-29, Colo320 and PC-3 cells were treated for 72 h with tubulysin A, **2**, **8** and **9**. SK-N-MC, MDA-MB-468, MDA-MB-231 and 184B5 cells were initially incubated with the peptide-toxin conjugate **8** for 6 h. After that initial incubation, the incubation solution was discarded, the cells were rinsed once with cell culture medium, and subsequently were allowed to proliferate in compound-free medium until 72 h were reached. Alternatively, SK-N-MC, MDA-MB-468, MDA-MB-231 and 184B5 cells were incubated for the whole experimental period of 72 h with the test items. Finally, resazurin solution in DMEM was added to yield a final resazurin concentration of 50 µM, and cells were incubated under standard growth conditions for 2 h. The conversion of resazurin to resorufin by viable, metabolically active cells was measured using a Synergy 2 multiwell plate reader (BioTek, Bad Friedrichshall, Germany) with 540 nm excitation and 590 nm emission filter setting. Non-linear regression analyses of these data was done by using GraphPad Prism software to calculate IC_{50} values.

Gene Expression Analysis

Samples for receptor expression analyses were prepared by RNA extraction using the RNA Mini Kit (Bio&Cell, Nürnberg/Feucht, Germany) and the RNeasy Mini Kit (Qiagen, Hilden, Germany), respectively, followed by a DNase I (Thermo Scientific/Fermentas, St. Leon-Rot, Germany) digestion step and cDNA synthesis

using oligo(dT) primers (Thermo Scientific, Schwerte, Germany) and RevertAid Premium Reverse Transcriptase (Thermo Scientific/Fermentas, St. Leon-Rot, Germany). All methods were done according to the manufacturer`s guidelines. The quality of the extracted RNA was checked by agarose gel electrophoresis and absorbtion spectroscopy. Quantitative PCR analysis was carried out using Bio-Rad`s CFX96™ real-time PCR detection system (CFX Manager™ Software 2.1) and SsoFast™ EvaGreen® Supermix (Bio-Rad, München, Germany), according to the manufacturer`s guideline. Briefly, each 20 µL PCR probe consisted of 10 µL master mix with 100 ng cDNA template and 8 pmol forward and reverse primer, respectively. The following customized primers (purchased from Thermo Scientific) were used for three reference genes: GAPDH (glyceraldehyde-3-phosphate dehydrogenase) – 5`-TTGCCATCAATGACCCCTTCA-3` (forward) and 5`-CGCCCCACTTGATTTTGGA-3` (reverse); HPRT (hypoxanthine-guanine phosphoribosyltransferase) – 5`-GGACAGGACTGAACGTCTTGC-3` (forward) and 5`-TGAGCACACAGAGGGCTACAA-3` (reverse); RPLP0 (60S acidic ribosomal protein P0) – 5`-GGCGACCTGGAAGTCCAACT-3` (forward) and 5`-CAATCAGCACCACAGCCTTC-3` (reverse). For the quantification of NPY1R (neuropeptide Y Y1 receptor subtype) expression levels the following customized primers (purchased from Thermo Scientific) were used: 5`-GCCGTTCCAAAATGTAACAC-3` (forward) and 5`-TGTTGTTTCTCCTTTTTAGGCG-3` (reverse).

The PCR setting was as follows: 30 s / 95 °C initial activation, 40 cycles of denaturation for 5 s / 95 °C and annealing for 5 s / 55 °C (plate read after each cycle), melt curve analysis between 65 – 95 °C with 0.5 °C increment for 5 s expression of

the receptor gene NPY1R was referenced to the house-keeping genes GAPDH, HPRT and RPLP0, and finally normalized to a reference cell line (e. g. MDA-MB-468 cells). Calculations were done using the formula $2^{(-\Delta\Delta Ct)}$ according to $\Delta\Delta C_t$ methodology.

Data Analyses

For data analyses GraphPad Prism version 5.03 and LibreOffice Calc versions 4.1.6.2 and 5.3.6.1 were used.

References

1. Pando, O.; Stark, S.; Denkert, A.; Porzel, A.; Preusentanz, R.; Wessjohann, L. A. *J. Am. Chem. Soc.* **2011**, *133* (20), 7692–7695.
2. Ahrens, V. M.; Kostelnik, K. B.; Rennert, R.; Böhme, D.; Kalkhof, S.; Kosel, D.; Weber, L.; von Bergen, M.; Beck-Sickinger, A. G. *J. Control. Release* **2015**, *209*, 170–178.
